# Font effects on reading parameters: comparing Radner Reading Charts printed in Helvetica and Times Roman

**DOI:** 10.1007/s00417-022-05665-y

**Published:** 2022-04-26

**Authors:** Wolfgang Radner, Michael Radner, Barbara Daxer, Thomas Benesch, Armin Ettl

**Affiliations:** 1 Karl Landsteiner University of Health Sciences, Dr. Karl-Dorrek-Straße 30, 3500 Krems, Austria; 2 Department of Ophthalmology, University Hospital St. Pölten, Dunant-Platz 1, 3100 St. Pölten, Austria; 3 Austrian Academy of Ophthalmology, Mollgasse 11, 1180 Vienna, Austria; 4 Institute for International Development, Sensengasse 3, 1090 Wien, Austria

**Keywords:** Reading charts, Standards of reading charts, Reading acuity, Equalized print sizes, x-height, Fonts effects on reading

## Abstract

**Purpose:**

To investigate the effect of font choice on reading parameters by using the RADNER Reading Charts printed in two fonts (Helvetica vs. Times Roman) equalized in terms of x-height.

**Methods:**

This is a cross-sectional study of 40 participants with healthy eyes (18 to 60 years of age; mean: 42.13 ± 12.28 years). Reading performance was evaluated binocularly with RADNER Reading Charts printed in either Helvetica Neue (T1) Roman sans serif (Adobe) or Times New Roman PS Roman serif (Adobe). The test distance was 40 cm. Reading charts were presented in random order. Reading acuity (RA), mean reading speed of all sentences read (MEAN-ALL RS), mean reading speed from 0.8 logRAD to 0.3 logRAD (MEAN-RS), maximum reading speed (MAX-RS), and critical print size (CPS) were compared.

**Results:**

The RA values obtained for the Helvetica and Times Roman fonts (in full logarithmic units of 0.1 logRAD) did not differ between the two fonts (mean for both fonts: − 0.128 ± 0.064 logRAD; 95% CI for both: − 0.148; − 0.107 logRAD). The differences in all other reading parameters between the two fonts were small and not statistically significant. The analyses revealed narrow confidence intervals and good coefficients of reliability. Except for the CPS (*r* = 0.49) and RA (equal for Helvetica and Times Roman), the correlations for all parameters were high, ranging from *r* = 0.92 to *r* = 0.98.

**Conclusion:**

The equivalent reading performance obtained with Helvetica and Times Roman (when equalized in x-height and layout) makes these font types interchangeable as standards for reading charts. 
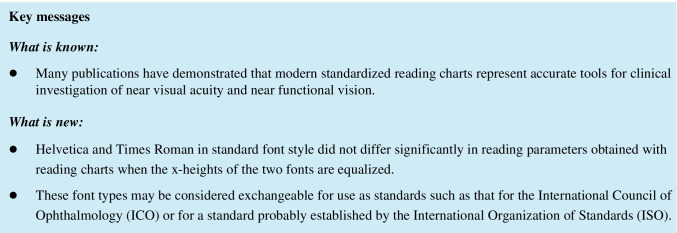

**Supplementary Information:**

The online version contains supplementary material available at 10.1007/s00417-022-05665-y.

## Introduction

In the past 20 years, many publications have demonstrated that standardized reading charts represent accurate tools for clinical investigation of near visual acuity and near functional vision [1–4]. Such reading charts have been developed in agreement with the requirements postulated by the International Council of Ophthalmology (ICO) [5] and provide geometrically (logarithmically) progressing print sizes [2, 3, 5]. In addition, some of them also allow an examination of further parameters of functional vision, such as reading acuity, maximum reading speed, mean reading speed, critical print size, reading acuity reserve, and reading speed based on reading acuity [1–3].

Although it seems evident that psychophysical tests must be standardized when used for diagnosis in human subjects, there are still many reading charts in use that do not adhere to the ICO or any other standard [6, 7]. This unfortunate situation has emerged because of the absence of an EN/ISO norm for reading charts analogous to that available for distance acuity charts [6–8]. In order to improve this undesirable situation, the committee for “Visual Optics and Instruments” of the International Organization of Standards (ISO) has recently approved a proposal to establish an ISO norm for reading and near vision charts and has installed a working group.

Although backgrounds for the definition of print sizes have been suggested by the already-existing ICO standard [6], there are several unanswered questions about the psychophysics of reading that need to be taken into account. One of these is whether there is an effect of typeface, particularly, serif vs. sans serif, on the results obtained with reading charts. In other words: Do different font types and font styles produce different results when both their “x-heights” (representing the middle height of a font type) and their layouts are equivalent?

As far as we are aware, three studies have dealt with a similar research question [9–11]. These studies have indicated that Courier and Times Roman differ slightly in several reading parameters[10] and that Helvetica and Times Roman do not differ in terms of reading speed [10, 11]. We have therefore had custom-made RADNER Reading Charts printed in Helvetica (as an example of a sans serif typeface) and Times New Roman (as an example of a typeface with serifs); the fonts in these charts had been equalized in x-height by means of a microscopic measuring system. In addition, the layout had also been equalized by a graphic designer. In the present study, we used these reading charts to determine whether these two font types differed in terms of reading acuity (RA), mean reading speed of all sentences read (MEAN-ALL RS), mean reading speed from 0.8 logRAD to 0.3 logRAD (MEAN-RS), maximum reading speed (MAX-RS), or critical print size (CPS).

## Methods

The study population of this prospective cross-sectional study consisted of 40 persons aged 18 to 60 (mean age: 42.13 ± 12.28 years; 22 women, 18 men). All subjects had come to the first author’s (ophthalmologist’s) outpatient facility for a routine eye check-up and/or for fitting of eyeglasses; in Austria, glasses are mainly prescribed by ophthalmologists. All of the participants had to be native speakers of German.

Participants were invited to participate in the study, and all who agreed gave informed consent. All of those who were eligible for the study were invited to participate. All study procedures adhered to the Declaration of Helsinki for research involving human subjects and the protocol for Good Scientific Practice (GCP). The study protocol was reviewed and approved by the ethics commission of the Karl Landsteiner University of Health Sciences.

The following routine procedures were performed prior to and after the study exam, as required for a routine check-up: anamnesis, visual acuity with and without glasses, orthoptic investigations, non-contact tonometry, and auto-refractometer measurements. The ophthalmologist (first author) examined the anterior segment with a slit-lamp. Then, subjective refraction (best corrected sphere and astigmatism) was evaluated, and reading performance was investigated. Participants were included if their decimal visual acuity was 1.0 or better (0.0 logMAR or better) in both eyes. The exclusion criteria were: optical correction in diopters outside the range of + 3.0 sphere (sph) + 1.5 cylinder (cyl) to − 6.0 sph + 1.5 cyl [12]; having any disease or receiving any medication that could influence the results of the study; having diabetes, pseudophakia, a history of stroke, an eye pressure above 20 mmHg; cataract (LOCS III): > NO2/NC2, C1, P0 [13]; any sign of amblyopia; corneal scars or signs of endothelial changes; a history of iritis or glaucoma; or any sign of retinal disease or optic nerve degeneration.

Participants 35 years of age or older were examined for presbyopia with RADNER Reading Chart 4 in order to identify the need for a near addition (reading glasses). If necessary, presbyopia was compensated by an adequate near addition. Participants read the study versions of the RADNER Charts either with their habitual (reading) glasses, when verified to be adequate, or with the actual best correction in test frames. This reading session was followed by Goldmann tonometry and fundoscopy. All participants were then invited to have a visual field test (Zeiss-Humphrey perimeter; 30.2 SITA fast) in order to exclude optic nerve or cerebral disease of the visual pathway. All study procedures were performed by the ophthalmologist (first author).

### Test material

**Fig. 1:**
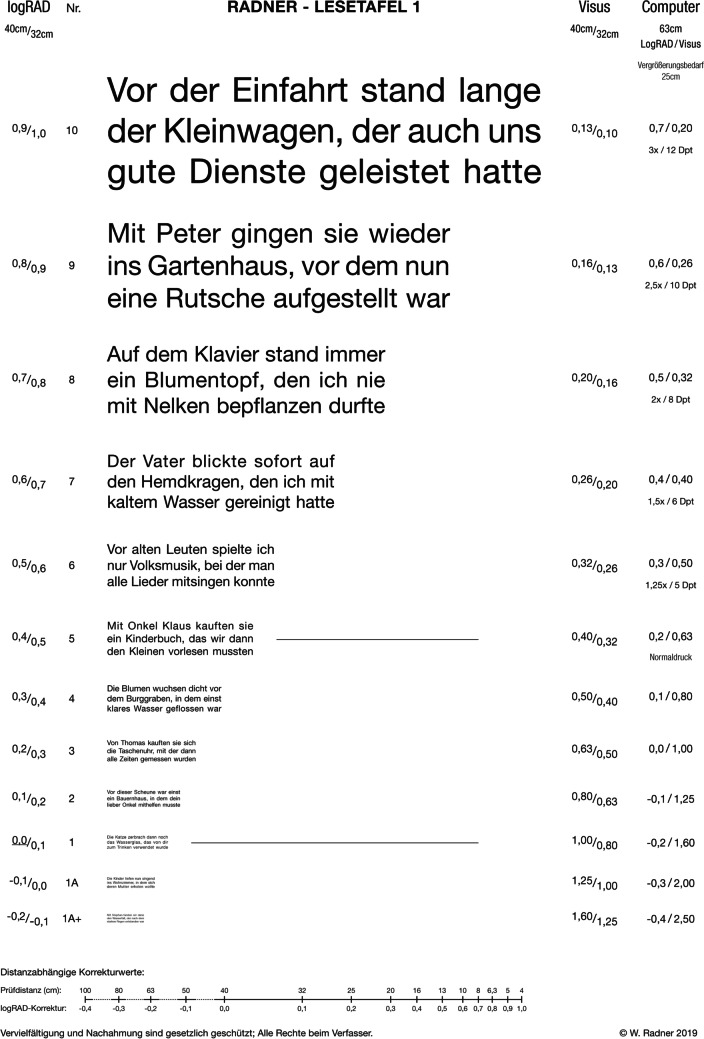
Radner reading chart 1 (orig. size: DIN A4)

**Fig. 2:**
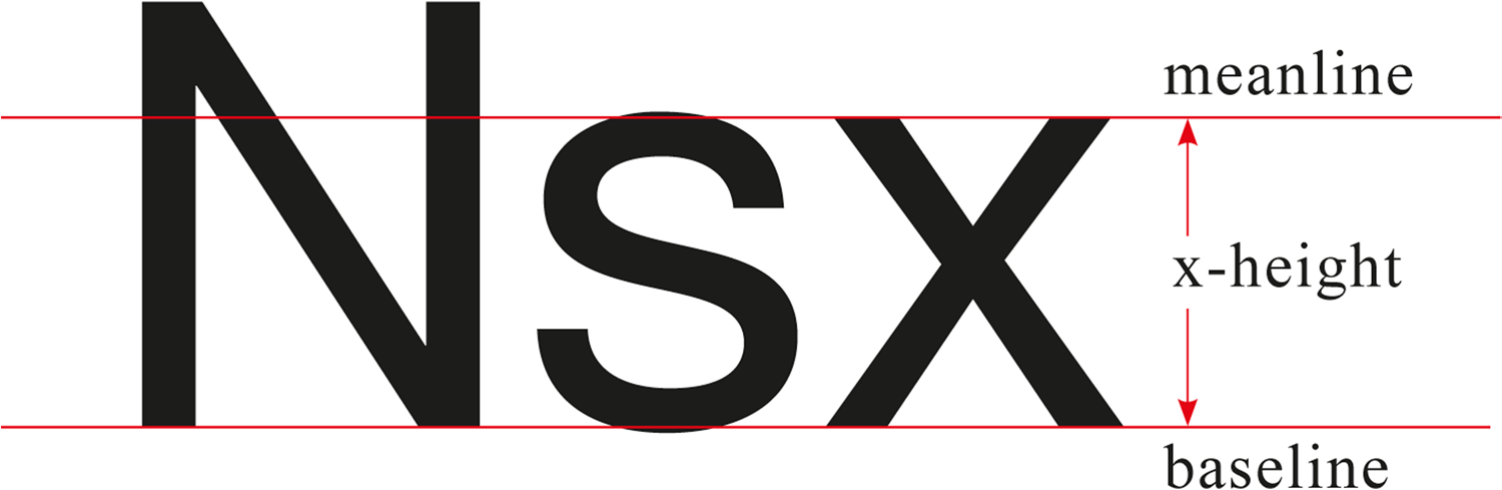
Graphic representation explaining the x-height: The x-height (height of the lowercase “x”) represents the distance between the baseline and the mean line for a font. Lowercase letters with round parts such as the “s” exceed these heights (overshoot these lines)

The test material was custom-made by a graphic designer. The Radner Reading Chart 1 and 2 (Fig. 1) were printed in Helvetica Neue (T1) Roman sans serif (Adobe) and in Times New Roman PS Roman serif (Adobe). The x-height was used as the reference measure for adjusting the print sizes of lower case letters of these fonts [5]. For each font, the x-height (the height of a lower case “x” in that font) is equal to the distance from the bottom of the letter”x” (indicated by the baseline) to the top of the same letter (indicated by the mean line) (Fig. 2). Both the layouts and the x-heights were equalized for the two fonts by the graphic designer by using a microscopic measuring system (NIS Elements, Nikon, Japan).

### Reading performance

Reading performance was investigated with RADNER reading charts 1 and 2 [14–16] printed in Helvetica Neue (T1) Roman sans serif (Adobe) and in Times New Roman PS Roman serif (Adobe). Evaluation of reading performance was performed binocularly with both versions of the RADNER Charts. The reading charts were presented to the probands in randomized order (patients pulled a card out of a box; on that card the pair of typefaces and the sequence of presentation were defined, so that every combination appeared with the same frequency). Reading acuity (RA), logRAD-score, critical print size (CPS), mean reading speed of all sentences read (MEAN-ALL RS), mean reading speed from 0.8 logRAD to 0.3 logRAD (MEAN-RS; six sentence optotypes), and maximum reading speed (MAX-RS) were analyzed (the definitions of the reading parameters are given in Table 1). The definitions of these reading parameters are given in Table 1. Reading acuity was measured by logReading Acuity Determination (log RAD), which represents the reading equivalent of logMinimal Angle of Resolution (log MAR)[2]. The logRAD is defined as the logarithm base 10 of the visual angle (minutes of arc) that subtends one-fifth of the x-height at the standardized distance of 40 cm. For example: For 0.0 logRAD at 40 cm the x-height is 0.582 mm. The visual angle for calculating the logRAD is calculated as: $60\bullet {\mathrm{tan}}^{-1}\left(\frac{0.582/5}{400}\right)=1.00\mathrm{ arc min}$.

**Table 1:** Definition of reading parameters

Parameter	Notation	Definition
RA^1^	logRAD (0.1 log-units)	logRAD = Reading equivalent of logMAR
logRAD Score^2^	logRAD	Sum of all syllables of misread words in the last sentence included × 0.005 + logRAD of this sentence
CPS^3^	logRAD (0.1 log steps)	Last print size read with normal reading speed (before a notable decrease in reading speed became apparent)
MEAN-ALL^4^	wpm	Average from all sentences read per reading chart
MEAN-RS^5^	wpm	Average from six sentences (0.8 logRAD to 0.3 logRAD)
MAX-RS^6^	wpm	Highest reading speed that could be achieved with one of the sentence optotypes read

^1^Reading acuity

^2^Reading acuity score

^3^Critical print size

^4^Mean of all sentences read

^5^Mean reading speed of six sentences read

^6^Maximum reading speed

The luminance was 100–110 cd/m^2^. The reading distance was determined with a 40-cm ruler and continuously verified during the procedure.

The reading charts were covered with a sheet of paper. The participants were instructed to uncover the text sentence by sentence and to read the sentences aloud as quickly and accurately as possible. They were further instructed to read to the end before correcting any reading errors. Measurements of reading time per sentence were performed with a stopwatch by considering the initial pre-movements of the lips at the vocal onset (pre-phonetic strain) as the starting point [15]. Reading speed in words per minute (wpm) was then calculated on the basis of the number of words (14 per sentence) and the reading time (accuracy: 0.01 s; reading speed = 840/reading time). Errors were counted even when immediately corrected. Stop criteria were: a reading time > 20 s (40 wpm); distortion of the content of the sentence; or more than 12 syllables read incorrectly.

### Statistics

The data showed a fairly symmetric unimodal distribution. The assumption of a normal distribution for the mean, as required for the *t* test, was justified (Kolmogorov–Smirnov). The cutoff level for statistical significance was set at a *p* value < 0.05 (two-tailed, paired). Pearson’s correlation was used to assess the association of variables. Statistical analyses were performed using SPSS for Windows software (version 21.0; IBM Corp.; Armonk, NY, USA).

## Results

The reading charts printed in Helvetica and Times Roman showed a high comparability and repeatability in all investigated parameters. Table 2 shows the mean, standard deviation, confidence interval, and coefficient of repeatability calculated for RA, logRAD-score, MEAN-ALL RS, MEAN-RS, MAX-RS, and CPS as obtained with RADNER Reading Charts printed either in either Helvetica or Times New Roman.

**Table 2:** Mean, standard deviation, confidence interval, and coefficient of repeatability

	Mean, SD, 95% CI for Helvetica and Times Roman		
	RA (logRAD) mean, SD, 95% CI	RA-Score (logRAD) mean, SD, 95% CI	CPS (logRAD) mean, SD, 95% CI
Helvetica	− 0.128 ± 0.06495% CI: − 0.148; − 0.107	− 0.11 ± 0.07595% CI: − 0.134; − 0.086	0.083 ± 0.05995% CI: 0.063;0.102
Times Roman	− 0.128 ± 0.06495% CI: − 0.148; − 0.107	− 0.11 ± 0.07495% CI: − 0.134; − 0.086	0.073 ± 0.072 95% CI: 0.050; − 0.095
	Mean-ALL RS (wpm) mean, SD, 95% CI	Mean-RS mean, SD, 95% CI	Max-RS mean, SD, 95% CI
Helvetica	170.18 ± 24.99 95% CI: 162.19;178.17)	202.01 ± 24.99 95% CI: 162.19;178.17)	223.09 ± 28.77 95% CI: 213.89;232.29
Times Roman	170.18 ± 21.53 95% CI: 163.29;177.06	200.86 ± 27.05 95% CI: 192.36;209.24	223.06 ± 31.69 95% CI: 212.92;233.19)

**Fig. 3:**
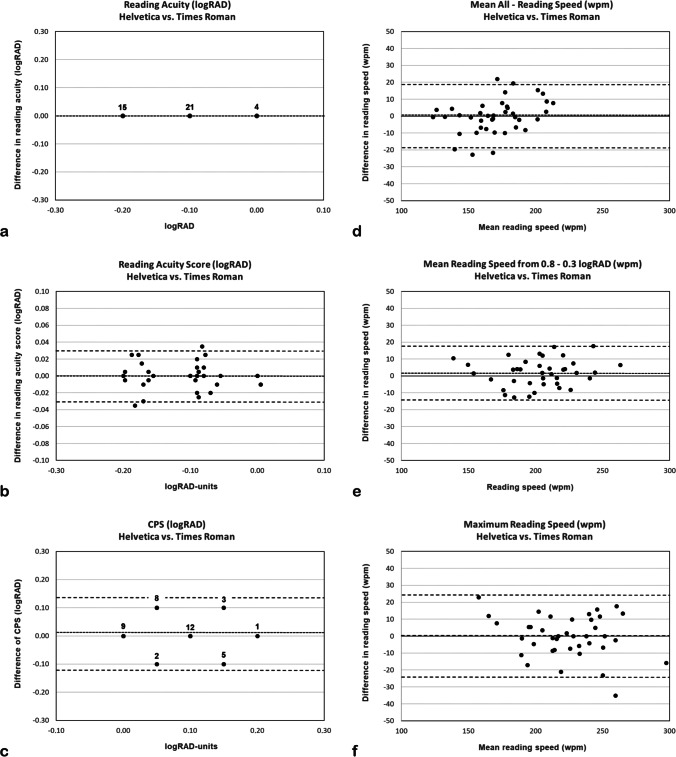
Bland–Altman plots comparing reading parameters obtained binocularly in 40 participants with healthy eyes reading RADNER Reading Charts printed in either Helvetica or Times Roman. (**a**) RA, (**b**) logRAD-score, (**c**) CPS, (**d**) MEAN-ALL RS, (**e**) MEAN-RS, and (**f**) MAX-RS

The analyses of the differences between the two chart versions with respect to these parameters are shown in Table 3 and by Bland–Altman plots (Fig. 3a–f). The differences in parameters that depend on reading acuity (RA, logRAD-score, CPS) and in those related to reading speed (MEAN-ALL RS, MEAN-RS, MAX-RS) were small, and they revealed narrow confidence intervals as well as good coefficients of reliability (Tables 2, 3, Fig. 3a–f). The parameter RA, which was obtained by applying the stop criteria in full logarithmic units of 0.1 logRAD, did not differ between fonts in any of the participants. Also, no significant differences between fonts could be found for any of the parameters (*p* values ranged from *p* = 0.10 to *p* = 1.00). Except for the CPS (*r* = 0.49) and the RA (equal values for Helvetica and Times New Roman), the correlations were high for all the parameters, ranging from *r* = 0.92 to *r* = 0.98.

**Table 3:** Differences between Radner Reading Charts

	Difference in logRAD—Helvetica vs. Times Roman					
	*t* test *p*	Corr.^1^ *r*	Mean Δ (logRAD)	SD Δ (logRAD)	95% CI Δ (logRAD)	CR Δ (logRAD)
RA	-	-	0.0	0.0	-	-
RA-Score	1.00	0.98	0.000	0.014	− 0.004; + 0.004	0.028
CPS	0.68	0.49	0.001	0.067	− 0.035; + 0.055	0.111
	Difference in wpm—Helvetica vs. Times Roman					
	*t* test *p*	Corr.^1^ *r*	Mean Δ (wpm)	SD Δ (wpm)	95% CI Δ (wpm)	CR Δ (wpm)
Mean-ALL RS	0.998	0.92	− 0.07	9.91	− 3.24; + 3.10	19.43
Mean-RS	0.10	0.96	2.13	7.89	− 0.40; + 4.65	15.46
Max-RS	0.86	0.92	0.03	12.14	− 3.85; + 3.92	23.79

^1^Correlation

## Discussion

In establishing a norm for reading charts, the possibility must be taken into account that font types could have an effect on the readability, and thus the comparability, of the results for reading parameters [9–11]. We have compared RADNER Reading Charts printed in either Helvetica or Times New Roman fonts that were custom-made to be equivalent in x-height and layout and have found a high comparability of RA, logRAD-score, CPS, MEAN-ALL RS, MEAN-RS, and MAX-RS between these two font types.

In the ICO standard, it is recommended that the print sizes progress geometrically and are based on the distance at which the height of lowercase letters such as “o,” “m,” and “x” subtends 5 min of arc [5]. For modern reading charts, using the x-height has become the preferred standard (Fig. 2), allowing a comparability of print sizes between different manufacturers [2, 3]. Another system for the definition of print sizes is the “point system” that originated from lead print and was applied to the “N” notation in 1951 [17]. However, point sizes are not related to the actual letter heights. They represent the size of the lead block on which the letters are mounted, so that letters of different font types vary in height even at a same point size. Thus, “N” can work only for one font type, which seems disadvantageous, given that preferences for font types change over time. In addition, the “point” is not an SI unit, which makes equalizing the letter heights of different font types complicated.

Calibrating the print sizes of font types with a Landolt ring would also be a possibility for standardizing print sizes. However, this would require visual acuity charts on which the Landolt rings are presented in adequate print quality. However, such reading charts are not available, since Landolt rings cannot be printed in sufficient quality below 0.0 logMAR [7]: Optotypes are smeared, resulting in lines that are too wide and openings in the Landolt rings that are too small, even in offset print. In contrast, offset-printed letters on reading charts exhibit a much higher print quality, because the offset technique has mainly been developed for printing text.

In addition, a number of objections can be raised with regard to possible study designs for calibrating reading charts with Landolt rings. For example, according to EN/ISO 8596, the distances between optotypes have to increase with smaller print sizes (the length of the acuity lines and the number of optotypes stay constant) [18]. In such cases, calibration would compare different visual tasks and crowding would be neglected. Thus, it seems evident that many technical challenges have to be overcome, and many aspects considered psychophysically before a calibration using Landolt rings can be considered a viable choice for standardizing reading charts.

Using the x-height for print size standardization seems to be the most suitable means of comparison, as was also implied by the results of the study by Rubin et al. [10]. They compared the reading speed obtained with four font types in participants with mild to moderate vision loss and found that Tiresias PC font (TPC) was read about 8 words min faster than the others. However, since in their case, fonts of the same nominal point size were not equivalent in actual size, the advantage of TPC was eliminated when the actual letter size and spacing were adjusted to be equivalent for the four fonts [10]. Our study supports this finding, in that we also could not find any significant differences in any of the investigated reading parameters between Helvetica and Times New Roman fonts for which the x-heights and the layout had been equalized.

Xiong et al. have compared five different font types by means of a tablet screen in order to determine whether there is an advantage associated with two new font types that have been developed for patients with maculopathy [11]. In their study, Helvetica and Times Roman were also compared. In agreement with the present study performed with printed reading charts, they could not find any significant difference in reading speed between these two font types in patients with maculopathy, in age-matched controls with healthy eyes, or 15 young subjects [11].

In the present study, there was no significant difference between RADNER Reading Charts printed in Helvetica or and those printed in Times New Roman in terms of any of the investigated reading parameters. Reading acuities obtained according to the standardized test protocol were equal. All other reading parameters, such as the RA score, which includes reading errors, revealed a high degree of similarity. Thus, these font types can be considered exchangeable for use as standards such as that for the ICO or for an upcoming ISO standard, assuming that the x-heights and layout are equalized.

## Supplementary Information

Below is the link to the electronic supplementary material.

Supplementary file1 (XLSX 17 KB)
